# Genetic Analysis of Stem Diameter and Water Contents To Improve Sorghum Bioenergy Efficiency

**DOI:** 10.1534/g3.120.401608

**Published:** 2020-09-09

**Authors:** Wenqian Kong, Huizhe Jin, Valorie H. Goff, Susan A. Auckland, Lisa K. Rainville, Andrew H. Paterson

**Affiliations:** Plant Genome Mapping Laboratory, University of Georgia, Athens, Georgia 30602

**Keywords:** Biparental QTL mapping, Genetic correspondence, GWAS, Sorghum, Stem diameter, Water content

## Abstract

Biofuel made from agricultural products has the potential in contribute to a stable supply of fuel for growing energy demands. Some salient plant traits, such as stem diameter and water content, and their relationship to other important biomass-related traits are so far poorly understood. Here, we performed QTL mapping for three stem diameter and two water content traits in a *S. bicolor* BTx623 x IS3620c recombinant inbred line population of 399 genotypes, and validated the genomic regions identified using genome-wide association studies (GWAS) in a diversity panel of 354 accessions. The discovery of both co-localized and non-overlapping loci affecting stem diameter traits suggests that stem widths at different heights share some common genetic control, but also have some distinct genetic influences. Co-localizations of stem diameter and water content traits with other biomass traits including plant height, flowering time and the ‘dry’ trait, suggest that their inheritance may be linked functionally (pleiotropy) or physically (linkage disequilibrium). Water content QTL in homeologous regions resulting from an ancient duplication event may have been retained and continue to have related functions for an estimated 96 million years. Integration of QTL and GWAS data advanced knowledge of the genetic basis of stem diameter and water content components in sorghum, which may lead to tools and strategies for either enhancing or suppressing these traits, supporting advances toward improved quality of plant-based biomass for biofuel production.

Stem diameter, specifically thicker stems and reduced lodging (Kashiwagi *et al.* 2008); and plant tissue water content, are important traits for cellulosic biofuel production. Given the advantages of sustainability and environmental friendliness, plant-based biofuel production has been envisioned by some to displace up to 30% of current U.S. petroleum consumption (Patzek and Pimentel 2005). The US Energy Information Administration projects a 48% increase in energy consumption between 2012 and 2040 (EIA 2016). Energy sources derived from renewable plant-based biomass have been suggested to be the only direct substitute for fossil fuels available on a significant scale and with less pollution (Tomes *et al.* 2011).

Plants having potential as biofuel feedstocks should require limited inputs (irrigation, fertilizer, pesticides), produce high yields of biomass, and be convertible to bio-based products in a relatively efficient and economical manner (Vermerris 2011). Leading candidate biofuel feedstocks include but are not limited to ‘conventional’ food cereals and oilseed food crops, *e.g.*, corn (*Zea mays*) starch and sugarcane (*Saccharum officinarum*) for ethanol, and soybean (*Glycine max*) for biodiesel; early ‘advanced’ non-food annual crops, *e.g.*, sorghum (*Sorghum bicolor*) starch and sugar for ethanol, and camelina (*Camelina sativa*) for biodiesel; perennial grasses and short-rotation forests, *e.g.*, Miscanthus (*Miscanthus* spp.) and poplar (*Populus* spp.) for lignocellulosic ethanol; and aquatic plants, *e.g.*, algae for biomass-derived biodiesel (Chaumont 1993; Tomes *et al.* 2011).

Being the fifth most widely grown cereal crop, sorghum was proposed as a potential biofuel feedstock over 30 years ago (Burton 1986). A C4 crop that converts energy into biomass more efficiently than C3 plants at warm temperatures, sorghum also has many advantages that make it an attractive biofuel feedstock, including lower need for fertilizers and pesticides than many crops, high water-use efficiency, drought tolerance, and wide adaptability to a variety of climates and soil conditions (Saballos 2008). In addition to its resilient nature, sorghum is also appealing for biofuel production for its short life cycle (4 months on average), abundant genetic diversity, and history of improvement of lignocellulose, sugar and starch yields. Efficient harvesting, storage, and transportation methodologies are in place, with various energy conversion options being explored (Rooney *et al.* 2007).

The per-acre yield and quality of plant biomass are important elements in the economics of plant-based biofuel production. Stem diameter, thicker being preferred for biomass yield, is itself a measurement to monitor plant growth. In sugarcane, a high correlation (r = 0.70) has been reported between stem weight and stem diameter (Milligan *et al.* 1990). In sorghum, a weak but significant correlation (r = 0.11) has been reported between sugar in dry stems and stem diameter (Murray *et al.* 2008). In sorghum, 13 stem diameter QTL have been reported on chromosomes 1, 2, 3, 4, 6, 7, 8, and 9 in a *S. bicolor* SS79 x M71 RIL population, using 157 AFLP, SSR, and EST-SSR markers (Shiringani *et al.* 2010). However, previous studies mostly measured stem diameter at only one location, *e.g.*, 5 cm above the ground in Miscanthus (Atienza *et al.* 2003), 20 cm above the ground in sorghum (Shiringani *et al.* 2010), or 40 cm above the ground in rice (Kashiwagi and Ishimaru 2004). By measuring and analyzing stem diameters at the base, midpoint and rachis, our study considers stem ‘shape’, and provides a more comprehensive investigation of QTL affecting stem architecture.

Plant water content, with less being preferred for biomass yield and storage (Gordon 1967), is associated with a variety of physiological mechanisms. Previous studies have indicated that this trait is plastic in nature and affected by many genes and/or environmental factors (Murray *et al.* 2008; Han *et al.* 2015). Some QTL have been reported in a number of sorghum mapping populations based on different measurement statistics. For example, 6 QTL for juice weight have been reported on chromosome 1, 4, 7 and 9 in a Shihong137 (grain sorghum) x L-Tian (sweet sorghum) cross, with the trait measured as the weight of stem juice squeezed using a sugarcane juice extractor within 24 hr of harvesting (Guan *et al.* 2011). A single QTL for relative juice weight was reported on chromosome 2 in a M71 (grain sorghum) x SS79 (sweet sorghum) RIL population, with the trait measured as wet stem weight - dry stem weight (Shiringani *et al.* 2010). One common drawback of these studies is that juiciness, conferred by a single gene (Hilson 1916; Xia *et al.* 2018; Zhang *et al.* 2018) that alters midrib morphology and tissue moisture level (Schertz *et al.* 1978), was not generally segregating widely in the study populations. For example, both parents of the RILs (Shiringani *et al.* 2010) were juicy – although transgressive segregation was observed, the power and accuracy of QTL detections could be reduced (Li *et al.* 2005). Water content, the percent difference between wet and dry biomass weight, may be a better estimate of plant moisture level than juice weight or relative juice weight, as it normalizes the moisture level to a scale of 0 to 1. Consistent with this definition, a major QTL (qSW6) for stem water content was reported on chromosome 6, accounting for 34.7–56.9% of the phenotypic variation at different internodes (Han *et al.* 2015). However, there is still a lack of knowledge about the genetic basis of leaf water content and the interaction between water contents of stems and leaves.

With the availability of genomic resources including genetic maps (Bowers *et al.* 2003), a high quality genome sequence (Paterson *et al.* 2009) and GBS SNP data (Morris *et al.* 2013) for a sorghum diversity panel (Casa *et al.* 2008), the genetic basis of stem diameter and water content traits can be further elucidated. Here, we couple biparental linkage mapping of quantitative trait loci (QTL) with genome-wide association studies (GWAS) taking advantage of long-term accumulation of historical recombination events, to discover the genetic basis of stem diameter and water content traits. The lower false-positive rate of QTL mapping and higher resolution of GWAS complement each other, providing valuable information for trait enhancement while mitigating constraints of each approach to accelerate gene mapping and identification (Tang *et al.* 2013).

In this study, we report QTL for three stem diameter traits and stem/leaf water contents in a *S. bicolor* BTx623 x IS3620c RIL population. These traits were further examined using GWAS in a sorghum diversity panel (Casa *et al.* 2008). The relationships of these traits to other important bioenergy traits, including plant height and flowering time are discussed. Identification of genomic regions responsible for stem diameter and water content traits can serve as a foundation for positional cloning of causal genes. Genomic regions identified here contribute to general knowledge of plant growth and development, with specific application toward genetic improvement of cultivars to produce biomass for biofuel production.

## Materials and Methods

### Genotypes

The genetic map of the *S. bicolor* BTx623 x IS3620c RIL population used for QTL mapping, as described (Kong *et al.* 2018), was constructed utilizing 399 individuals and 616 genotyping-by-sequencing (GBS) based SNP markers. It collectively spanned 1,404.8 cM on 10 linkage groups with a 3.8 cM average interval between consecutive markers.

The genotypes for genome-wide association study (GWAS) were generated for a US sorghum diversity panel (Casa *et al.* 2008), including a total of 265,487 SNPs in 27,412 annotated genes across 354 sorghum accessions (Morris *et al.* 2013).

### Phenotypes

Phenotypic data (File S1) for QTL mapping was measured for the *S. bicolor* BTx623 x IS3620c RIL population in 2011 and 2012, with single 3 m plots of 10-15 plants grown in completely randomized designs in each year. The RIL population was planted on May 10^th^, 2011 and May 18^th^ 2012. Phenotypic data for GWAS was measured for the sorghum diversity panel in 2009 (seeds sowed on May 19^th^) and 2010 (seeds sowed on May 26^th^) as described by Zhang *et al.* (2015). For both populations, plants were harvested when the main heads of a genotype reached senescence. Both populations were grown at the University of Georgia Plant Science Farm near Watkinsville GA (33°52’28.1”N, 83°31’37.2”W). Three stem diameters (base, middle, rachis) and four plant weights (wet stem weight, dry stem weight, wet leaf weight, dry leaf weight) were recorded for two plants (as subsamples) per plot with panicles removed. Stem diameters were measured using calibrated digital calipers at the thickest point of the indicated locations. The middle of a plant was determined by dividing the length from the base to the rachis by two. Fresh weights of leaves and stalks were measured at physiological maturity, with dry weights measured after drying to stable mass in a tobacco barn. The water contents of stems and leaves were defined as: (weightwet−weightdry)weightwet× 100%. Pearson correlation coefficients were calculated between traits of interest.

### Heritability

Broad-sense heritabilities for stem diameter and water content traits were calculated based on the impact of genotype (G), environment (E) and genotype by environment interaction (G X E), using the lme4 library of R version 3.2.3 (Bates *et al.* 2015). Different years (2009 and 2010) were treated as different environments. The mixed model utilized is:

y=Xβ+Zμ+e

where genotype, environment and their interactions are all considered random factors. Variance components used to calculate heritability were determined by the restricted maximum-likelihood (REML) method, with their significance estimates tested by model comparison with likelihood ratio tests (Longin and Wurschum 2014). Broad-sense heritability was then calculated as: $H=var_{Line}/(var_{Line}+\frac{var_{Line\times Year}}{E}+\frac{var_{Residual}}{ER})$, in which E is the number of environments and R is the number of subsamples per plot (Kong *et al.* 2014).

### QTL mapping

Overall BLUP (Best Linear Unbiased Prediction) values used to detect QTL were calculated for each line using the mixed model described above (RCoreTeam 2015). For each trait, potential QTL were detected by interval mapping using the R/qtl Package (Broman *et al.* 2003), with a LOD (logarithm of the odds ratio) threshold of 2.5. Significant QTL detected were then considered as fixed effects to scan for additional QTL. Then, all potential QTL were used to fit an additive QTL model (Arends *et al.* 2010). Backward selection was then performed to exclude QTL below the threshold (2.5). Then, we used the ‘refineqtl’ function to determine the optimum position and effect for QTL. The proportions of variation explained by QTL were then calculated from the final additive model. Physical locations of QTL were delineated by anchoring to the reference genome the two flanking markers nearest to the 1-LOD interval boundaries that have alignment information as described (Zhang *et al.* 2013), based on colinearity between genetic and physical marker positions. The LOD threshold 2.5 used in this analysis is slightly less restrictive than the threshold from permutation tests (2.95 and 2.66 for 5% and 10% significance levels, respectively).QTL nomenclature is as described by McCouch *et al.* (1997), starting with a lowercase ‘q’ followed by abbreviations of trait names in capital letters, then the year (if not for overall BLUP values, optional), chromosome number, and a decimal numeric identifier to differentiate multiple QTL on the same chromosome.

### GWAS

Genome-wide association studies were conducted using 265,487 published SNPs (Morris *et al.* 2013) for a sorghum diversity panel (Casa *et al.* 2008) and trait data that we collected in 2009 and 2010. GWAS was conducted using a compressed Mixed Linear Model (cMLM), which took into account a genetic marker-based kinship matrix and a principal component-based population structure term (Zhang *et al.* 2010). The model selection feature of Genomic Association and Prediction Integrated Tool (GAPIT) (Lipka *et al.* 2012) was used to determine the compression level and the optimal number of principal components (Zhu and Yu 2009). To ensure the quality of GWAS, log quantile-quantile (QQ) plots were used to monitor systematic sources of spurious associations. To determine the significance threshold for GWAS, a Bonferroni-like multiple testing correction (Matthies *et al.* 2014; Zhang *et al.* 2015) was used rather than the traditional Bonferroni method. The traditional Bonferroni method is too stringent to detect QTL which may reduce power to detect true associations. To balance an acceptable false positive rate with sufficient detection power, we integrated LD-information for each chromosome in determining the significance threshold as $\alpha /(\underset{i}{\sum }\frac{l_{i}}{d_{i}})$, where $l_{i}$ is the length of chromosome i, $d_{i}$ is the extension of LD for chromosome i, which is the distances in kilobases until linkage disequilibrium decays to r^2^ < 0.1 for each chromosome in the sorghum diversity panel [found in Table S1 of Morris *et al.* (2013)], and α=0.05 is the genome-wide significance threshold for all tests. As a result, a P-value of 1.96×10^−5^ (0.05/2552.72) was used as the significance threshold for GWAS. Details of the chromosome length and the extension of LD can be found in Table S1.

### Reference genomes

*Sorghum bicolor* gene annotations refer to JGI annotation release Sbi1.4 (Paterson *et al.* 2009).

### Data availability

Phenotypic data are accessible through figshare. Genotypic data of the BTx623× IS3620C is available at figshare: https://doi.org/10.25387/g3.6304538. Genotypic data of the GWAS analysis is available through https://www.morrislab.org/data. The authors affirm that all data necessary for confirming the conclusions of the article are present within the article, figures, and tables. Supplemental material available at figshare: https://doi.org/10.25387/g3.12805073.

## Results

### Phenotypic distribution

Descriptive statistics for the three stem width variables: basal stem diameter (BD), middle stem diameter (MD), and rachis diameter (RD); four weight variables: wet stem weight (WSW), dry stem weight (DSW), wet leaf weight (WLW), and dry leaf weight (DLW); and two derived water-content variables: stem water content (SWC), leaf water content (LWC), of the sorghum diversity panel (Casa *et al.* 2008) and BTx623 x IS3620c RILs are shown in Table S2. Plant water content is normally higher with less variation in stems (SWC) than leaves (LWC). Differences in means and ranges between years reflect environmental effects (Table S2).

Correlation coefficients among these 9 traits (Table S3) in the sorghum diversity panel and BTx623 x IS3620c RILs suggest many relationships in each of the two populations. Significant correlations among three stem variables: BD, MD, RD (Table S3, correlation in sorghum diversity panel/ BTx623 x IS3620c RILs: r_BD:MD_ = 0.60/0.57, r_BD:RD_ = 0.56/0.68, r_MD:RD_ = 0.72/0.72, all p-values < 0.0001); four weight variables: WSW, DSW, WLW, DLW (Table 2, r_DSW:WSW_ = 0.92/0.95, r_DSW:DLW_ = 0.74/0.85, r_DSW:WLW_ = 0.58/0.75, r_WSW:DLW_ = 0.80/0.85, r_WSW:WLW_ = 0.66/0.80, r_DLW:WLW_ = 0.93/0.93, all p-values < 0.0001), and two water-content variables: SWC, LWC (Table S3, r_SWC:LWC_ = 0.21/0.48, p-value < 0.0001) are observed in both populations. Positive correlations between stem diameter (BD, MD, RD) and biomass yield (DSW, DLW) are verified. There were positive correlations of SWC with BD, RD; and LWC with WLW, DLW; and negative correlation of SWC with DSW in both populations. The significant correlation of LWC and RD was negative (Table S3, r_LWC:RD_ = -0.09, p-value = 0.0200) in the sorghum diversity panel but positive (Table S3, r_LWC:RD_ = 0.13, p-value < 0.0001) in the BTx623 x IS3620c RILs. However, given the large sample sizes used to calculate the correlation coefficients (n1 = 354×2, n2 = 393× 2), even very low correlation coefficients are significant.

### Heritability

Variance component estimates and heritability estimates (Table S4) for stem diameter and water content traits were similar in the two populations. The genotypic effects are statistically significant (p-value < 0.001) for all traits except SWC in the sorghum diversity panel (Table S4a), where only the environmental effect is significant (p-value < 0.001). The observation that genotype is not a significant determinant of SWC in the diversity panel could be due to stem wet and dry weights being highly variable among years (Table S2a). For stem diameter variables (BD, MD, RD), genotype by environment interactions are statistically significant (p-value < 0.001) in both populations. Their heritabilities were moderate-high, ranging from 0.55 to 0.70, similar to what was reported in *S. bicolor* M71 x SS79 RILs (h = 0.60) (Shiringani *et al.* 2010). Heritability for water content variables ranges from 0.25 to 0.39, due to larger environmental effects (Year) than for stem diameter traits in both populations, possibly caused by variation in harvest time. In consideration of different levels of plasticity among traits, we adopt both overall BLUP values since genotype explains a large amount of total variances (see Materials and Methods) across environments (years) and single year values of the traits to detect environment specific QTL signals.

### QTL results

#### QTL for stem diameter traits:

**Figure 1:**
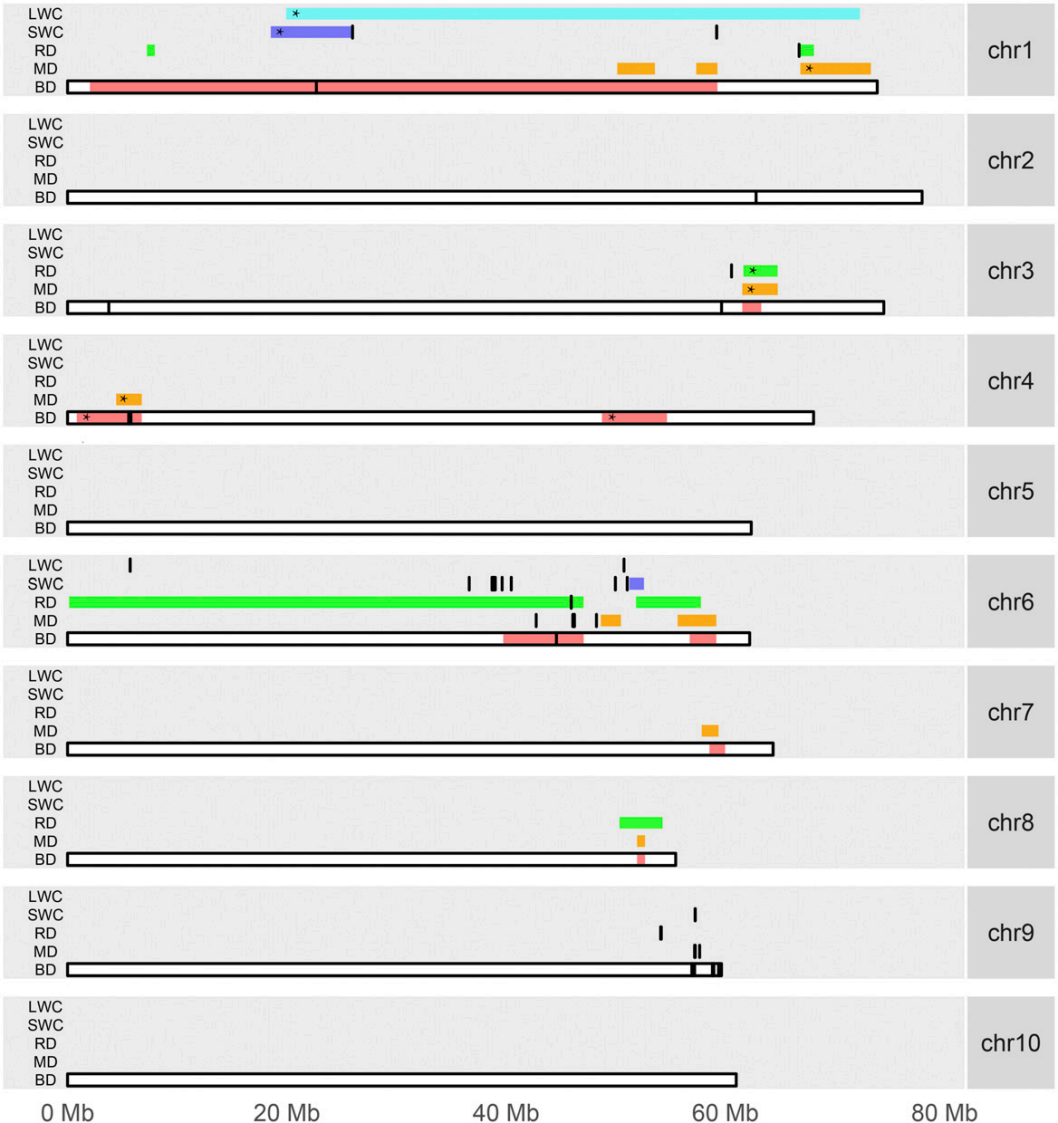
QTL and significant SNPs for stem diameter and water content traits along the *S. bicolor* reference genome (Paterson *et al.* 2009). 1-LOD intervals of QTL in BTx623 x IS3620c RILs for BD (red), MD (orange), RD (green), SWC (purple) and LWC (blue) are shown. QTL intervals derived using single year values are marked with asterisks. SNPs significantly associated with traits in a sorghum diversity panel (Casa *et al.* 2008) at a threshold of 1.96×10^−5^ are shown as solid lines. BD, basal stem diameter; MD, middle stem diameter; RD, rachis diameter; SWC, stem water content; LWC, leaf water content.

Totals of 6 QTL affecting basal stem diameter (on chromosomes 1, 3, 6 [2], 7, 8), 6 QTL affecting middle stem diameter (on chromosomes 1 [2], 6 [2], 7, 8), and 5 QTL affecting rachis diameter (on chromosomes 1 [2], 6 [2], 8) were detected based on overall BLUP values, respectively explaining 28.9%, 26.0% and 20.0% of phenotypic variation in additive QTL models (Table 1 and Figure 1). Additional environment-specific QTL (Table S5) that do not overlap with those from overall BLUP values include 2 for BD (on chromosome 4), 3 for MD (on chromosomes 1, 3, 4), and 1 for RD (on chromosome 3), reflecting genotype x environment interactions.

**Table 1:** QTL affecting stem diameter and water content traits using overall BLUP values in BTx623 x IS3620c RILs

Trait	QTL name	Chr.	Peak (cM)	LOD	Additive Effect *^a^*	R^2^ (%)	Start (Mb)*^b^*	End (Mb)*^b^*
BD	qBD1.1	1	67.0	3.54	0.82	3.01	1.9	59.4
	qBD3.1	3	113.8	4.83	−0.76	4.14	61.4	63.4
	qBD6.1	6	32.0	5.97	0.85	5.15	39.6	47.2
	qBD6.2	6	93.0	8.18	−1.00	7.16	56.6	59.3
	qBD7.1	7	97.0	4.41	−0.72	3.77	58.4	60.1
	qBD8.1	8	83.0	6.79	−0.92	5.88	51.8	52.8
MD	qMD1.1	1	96.5	3.85	0.42	3.43	50.0	53.7
	qMD1.2	1	124.9	5.25	−0.48	4.67	57.2	59.4
	qMD6.1	6	51.0	2.76	−0.35	2.44	48.5	50.6
	qMD6.2	6	82.0	2.54	−0.31	2.27	55.5	59.3
	qMD7.1	7	87.0	8.76	0.60	8.07	57.7	59.5
	qMD8.1	8	83.3	5.61	−0.48	5.01	51.8	52.8
RD	qRD1.1	1	25.7	3.97	−0.36	4.03	7.1	8.1
	qRD1.2	1	160.5	7.57	−0.36	7.02	66.7	68.2
	qRD6.1	6	4.0	3.12	0.26	3.24	0.0	47.2
	qRD6.2	6	69.0	3.78	−0.26	3.82	51.7	57.9
	qRD8.1	8	84.0	2.77	−0.24	3.09	50.2	54.4
SWC	qSWC6.1	6	59.0	20.24	−0.0127	20.86	51.1	52.7

BD basal stem diameter, MD middle stem diameter, RD rachis diameter, SWC stem water content.

a Additive effects calculated as IS3620c – BTx623.

b Based on DNA marker locations flanking 1-LOD intervals in the published genome sequence (Paterson *et al.* 2009).

Overlapping QTL regions for BD, MD and RD (Table 2a, Figure 1) based on overall BLUP values were found on chromosomes 3, 6 and 8. In addition, 4 overlapping QTL regions for BD and MD were observed on chromosomes 1, 4, and 7 (Table 2b); 2 overlapping QTL regions for BD and RD were observed on chromosomes 1 and 6 (Table 2c); and 1 overlapping QTL region for MD and RD was observed on chromosome 1 (Table 2d).

**Table 2:** Overlapping QTL regions among different measures of stem diameter

a) BD, MD and RD					
Chr.	BD	MD	RD	Start (Mb)	End (Mb)
3	qBD3.1	qMD12_3.1*^a^*	qRD12_3.1*^a^*	61.5	63.4
6	qBD6.2	qMD6.2	qRD6.2	56.6	57.9
8	qBD8.1	qMD8.1	qRD8.1	51.8	52.8
					

b) BD and MD					
Chr.	BD	MD	Start (Mb)	End (Mb)	
1	qBD1.1	qMD1.1	50.0	53.7	
1	qBD1.1	qMD1.2	57.2	59.4	
4	qBD12_4.1*^a^*	qMD12_4.1*^a^*	4.3	6.9	
7	qBD7.1	qMD7.1	58.4	59.5	

c) BD and RD					
Chr.	BD	RD	Start (Mb)	End (Mb)	
1	qBD1.1	qRD1.1	7.1	8.1	
6	qBD6.1	qRD6.1	39.6	47.2	

d) MD and RD					
Chr.	MD	RD	Start (Mb)	End (Mb)	
1	qMD11_1.1*^a^*	qRD1.2	66.7	68.2	

BD basal stem diameter, MD middle stem diameter, RD rachis diameter.

a QTL based on single year values (qMD12 indicate data from 2012).

#### QTL for water content traits:

One QTL affecting SWC were detected on chromosome 6 based on BLUP data, explaining a 20.24% of total phenotypic variance. Two additional environment-specific QTL were found on chromosomes 1 (from 2012 data only) and 6 (2012), explaining 3.3% and 3.0% total variances, respectively. Only one QTL affecting LWC on chromosome 1 (only from 2012 data) were detected, explaining 3.0% of phenotypic variation respectively (Table S5, Figure 1). There were no QTL detected for LWC based on overall BLUP values, but one QTL was detected based on single year values from 2011 (Table S5).

#### Overlap of stem diameter and water content QTL to those identified in other studies:

A previous study (Shiringani *et al.* 2010) reported 13 QTL for stem diameter on chromosomes 1, 2, 3, 4, 6, 7, 8, and 9 in a *S. bicolor* SS79 x M71 RIL population, with the trait measured 20 cm above the ground. We determined the physical locations (Table S6) of a total of seven QTL by aligning markers flanking the support intervals to the reference genome (Paterson *et al.* 2009) with four QTL corresponding to those found in our study. Most QTL reported by Shiringani *et al.* that were not found in our QTL mapping were found by GWAS (below, except the one on chromosome 8), which is a good complement to biparental linkage mapping. A QTL for rachis diameter was reported in a subset of 119 lines from our *S. bicolor* BTx623 x IS3620c RIL population that was also detected in our study, located on chromosome 6 (qRD6.1, 3Mb to 49 Mb) (Brown *et al.* 2006). Curiously, both our study and the prior one (Brown *et al.* 2006) found that qRD6.1 for RD and the co-localized qBD6.1 for BD have effects in the opposite direction of what would be expected based on parental phenotypes (Table 1), the BTx623 allele being associated with reduced stem diameter although BTx623 has much thicker stems than IS3620c.

One overlapping QTL region detected for SWC and LWC on chromosome 1 from 2012 data (Table 3, Table 4, Figure 1) also overlapped with a QTL for BD (qBD1.1) (Table 4). There is another overlapping QTL region detected for SWC (qSWC6.1) and RD (qRD6.2) on chromosome 6 (Table 4). These two QTL clusters, which may contribute to inter-relationship between traits, were supported by significant correlations in the mapping population among BD, SWC, and LWC (Table S3b), and between RD and SWC (Table S3b). They could be either due to pleiotropic effects of single genes, or to proximal locations of different genes related to stem width and water content. In comparison to prior studies, qSWC6.1 was near a previously reported stem moisture locus qWC6 (Han *et al.* 2015) in a Shihong137 (grain sorghum) x L-Tian (sweet sorghum) cross. Further, qSWC12_1.1 was in a region partly homeologous to qSWC6.1 (Table S8), but eluded detection in the previous report.

**Table 3:** Stem diameter and water content QTL with corresponding significant SNPs and overlap with *Dw* or *Ma* genes

Trait	QTL	Chr.	Significant SNPs	Distance*^a^*	Plant height or flowering genes
BD	qBD1.1	1	S1_22691388	Within*^b^*	EHD1
BD	qBD3.1	3	S3_59642849	1.8Mb	
BD	qBD12_4.1	4	S4_5581387, S4_5581390, S4_5774418	Within*^b^*	
BD	qBD6.1	6	S6_44581098	Within*^b^*	
MD	qMD6.1	6	S6_42736415, S6_46077506, S6_46194160, S6_46217845, S6_46217893, S6_46217966, S6_48234025	0.3Mb	Dw2
RD	qRD1.2	1	S1_66715300	Within*^b^*	
RD	qRD12_3.1	3	S3_60554780	1Mb	
RD	qRD6.1	6	S6_45935408	Within*^b^*	
SWC	qSWC12_1.1	1	S1_25989043	Within*^b^*	
SWC	qSWC6.1	6	S6_49958866, S6_49958867, S6_51042333	60kb	

BD basal stem diameter, MD middle stem diameter, RD rachis diameter, SWC stem water content, LWC leaf water content.

a Distance between QTL to the nearest significant SNP outside the QTL interval.

b Significant SNPs are contained in the QTL interval.

**Table 4:** Overlapping QTL regions between water content and stem diameter traits

Chr.	SWC	LWC	BD	RD	Start (Mb)	End (Mb)
1	qSWC12_1.1*	qLWC12_1.1*	qBD1.1		19.8	26.2
6	qSWC6.1			qRD6.2	51.7	52.7

BD basal stem diameter, RD rachis diameter, SWC stem water content, LWC leaf water content.

* QTL based on single year values.

### GWAS results

#### Stem diameters:

**Figure 2:**
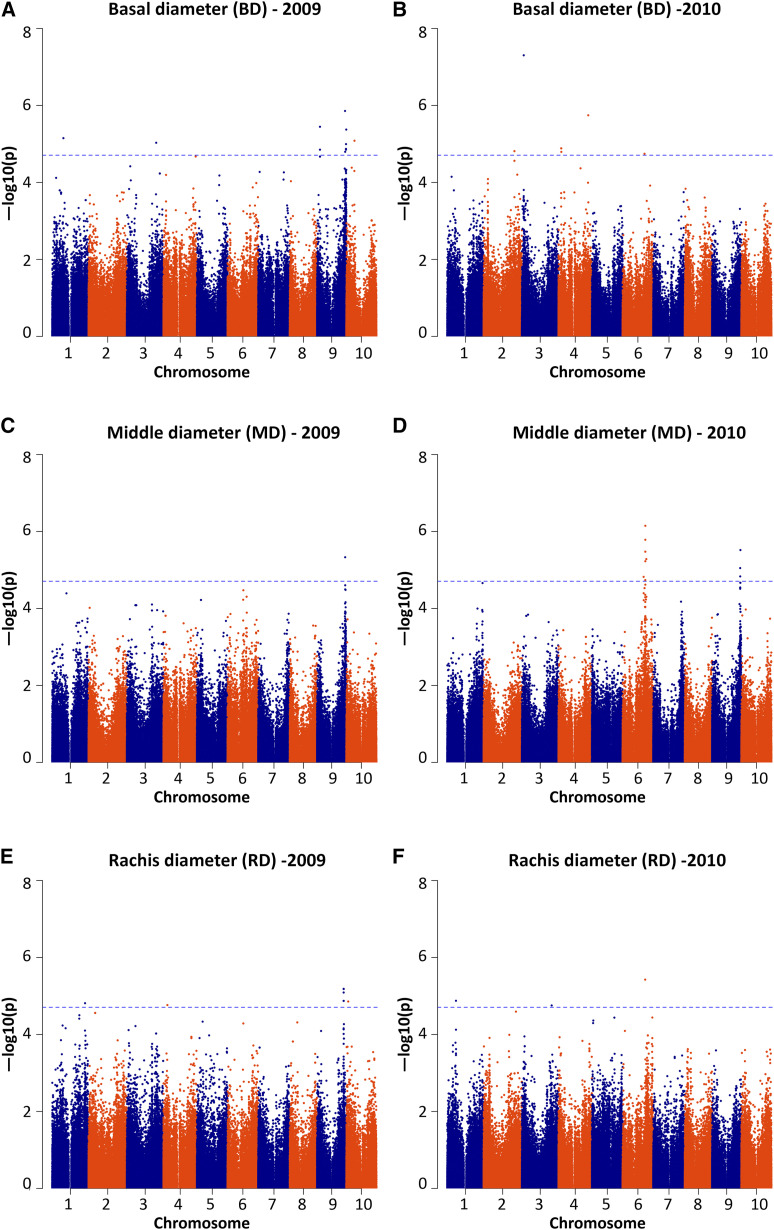
Manhattan plots for stem diameter traits, including basal (a, b), middle (c, d) and rachis (e, f) diameter, in a sorghum diversity panel (Casa *et al.* 2008). An experiment-wise significance threshold of 0.05 adjusted for multiple comparisons [-${\text{log}}_{10}$(p-value)∼4.7] is noted with dashed horizontal lines.

A total of 33 SNPs (14 for BD, 10 for MD, 9 for RD) (Figure 1, Figure 2; Supporting Information Table S7) of common variants (minor allele frequency ≥ 5%) were significantly associated with stem diameter traits, with no confounding by population stratification (Figure S1). A few significant SNP markers were considered potentially as false positives and were removed based on marker locations and linkage disequilibrium, since spurious associations tend to stand alone (*i.e.*, with no nearby SNPs showing association). Few significant associations were shared between the two years, implying a large role of environmental factors (Table S1) (Manolio *et al.* 2009).

**Figure 3:**
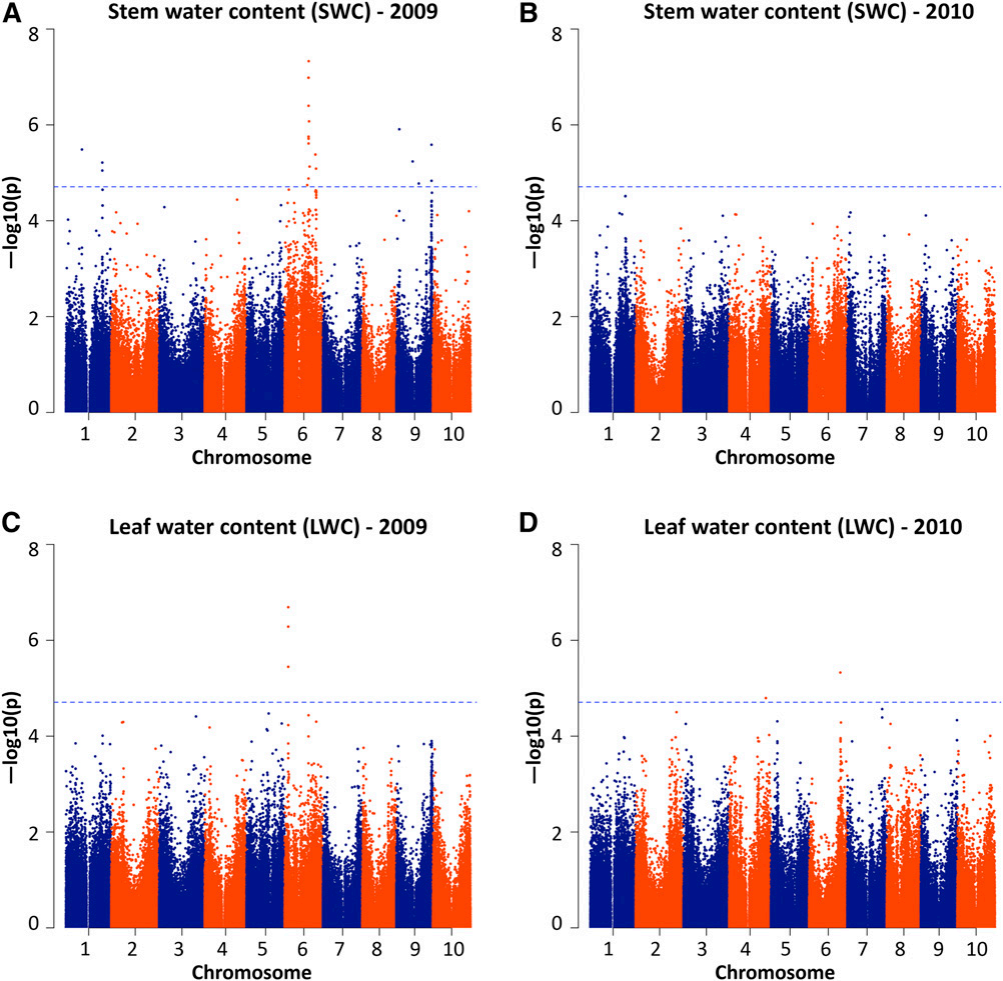
Manhattan plots for water content traits, including stem water content (a, b) and leaf water content (c, d), in a sorghum diversity panel. An experiment-wise significance threshold of 0.05 adjusted for multiple comparisons [-${\text{log}}_{10}$(p-value)∼4.7] is noted with dashed horizontal lines.

To compare the results of GWAS and QTL mapping for stem diameter, we grouped the SNPs based on their locations (Figure 1, Table 3) Significant SNPs for BD, MD and RD were closely associated with QTL on chromosomes 1, 3, 4 and 6. Other significant SNPs (on chromosomes 2, 3 and 9) that were not closely associated with our QTL were associated with previously reported QTL (Shiringani *et al.* 2010). SNPs on chromosome 9 suggested a fourth overlapping genomic region (54.1Mb to 59.4Mb) for BD, MD and RD; while associations on chromosomes 2 (peak at 62.8Mb) and 3 (peak at 3.8Mb) were detected in 2010 only, suggesting environment-specific loci for BD. This provides further support to our hypothesis that stem widths at different heights (base, middle, rachis), share some common genetic control but also have some distinct genetic influences (Figure 3).

## Discussion

The *S. bicolor* BTx623 x IS3620c RIL population offers opportunities to study the genetic control of stem diameter and water content traits in sorghum. Linkage mapping validated the positions and effects of several previously detected QTL, provided evidence for novel QTL that eluded detection in prior studies, and provided new insights into patterns of genetic control of stem diameter traits by taking advantage of multiple measurements at different stem heights. GWAS using a sorghum diversity panel (Casa *et al.* 2008) complemented linkage mapping by providing support to many QTL detected and indicating multiple novel putative loci that eluded detection from linkage mapping in the study population but are known from prior studies. The high resolution of GWAS can aid in identification of causative loci by targeted re-sequencing of genes surrounding the peak of associations. Together with other resources beyond the QTL meta-data and GWAS comparative data that we have used here, *e.g.*, examining expression profiles in particular tissues, one can envision a practical path to the identification of small numbers of high-probability candidate genes.

Among the three stem diameter variables studied, basal diameter is consistently positively correlated with water contents in stems and leaves. From a biofuel perspective, however, exceptional genotypes in which basal stem diameter is associated with reduced water content would be preferred. Co-localizations of loci affecting stem diameter and water content traits with other biofuel-related traits were also observed. On chromosome 6, there is a cluster of associations (39.6Mb to 47.2Mb) for basal stem diameter (qBD6.1, association peak at S6_44581098), middle stem diameter (association peak at S6_45935408), rachis diameter (qRD6.1, association peak at S6_45935408), plant height *Dw2* (Morris *et al.* 2013), flowering time *Ma6* (Brady 2006) and primary branching number (Kong *et al.* 2014; Brown *et al.* 2006). On chromosome 9, there is a cluster of associations (54.1Mb to 59.4Mb) for basal stem diameter (association peak at S9_56928114), middle stem diameter (association peak at S9_57240634), rachis diameter (association peak at S9_54137819), plant height and flowering time (Zhang *et al.* 2015). By investigating several stem diameters distributed over the plant rather than a single measurement, our study provides more information than prior studies about patterns of genetic control. For example, the 2 overlapping QTL regions for BD, MD and RD (Table 2a) on chromosomes 6 and 8; 3 overlapping QTL regions for BD and MD (Table 2b) on chromosomes 1 and 4; and 1 overlapping QTL region for MD and RD (Table 2d) on chromosome 1 that we report, eluded detection in previous studies.

The ‘dry’ trait is the phenotype of a dry white midrib as opposed to a juicy green midrib controlled by a recently cloned single gene *Dry* (Zhang *et al.* 2018; Xia *et al.* 2018). The *Dry* allele often occurs in grain sorghum while the juicy one in sweet sorghum. With regard to water content traits, there is co-localization of stem water content qSWC6.1 and the *Dry* gene for the ‘dry’ trait (Hart *et al.* 2001; Xu *et al.* 2000), which was identified on sorghum chromosome 6 and has been recently cloned to reduce plant water content (Zhang *et al.* 2018; Xia *et al.* 2018). The ‘dry’ phenotype is manifested by white leaf midribs and dry, pithy stalks, which influences SWC in this study. GWAS identifies a locus (peak at 50.7Mb) very close to the D gene (<1Mb), complementing QTL mapping, especially for traits of high plasticity. These co-localizations of bioenergy traits indicate that their inheritance may be linked functionally (pleiotropy) or physically (linkage disequilibrium). In addition, qSWC12_1.1 and qSWC6.1 are in homeologous regions resulting from an ancient duplication event (Table S8). This suggests that paleo-duplicated gene pairs affecting stem water content may have been retained and continue to have related functions for an estimated 96 million years following this event (Wang *et al.* 2015), albeit being substantially influenced by the environment.

In conclusion, this study identified the genetic basis for three stem diameter variables and two water content variables in sorghum by using a combination of linkage mapping and genome-wide association mapping approaches. QTL for basal stem diameter, middle stem diameter (on chromosomes 1, 3, 4, 6, 7, 8), rachis diameter (on chromosomes 1, 3, 6, 8), stem water content (on chromosomes 1, 6), and leaf water content (on chromosome 1) were reported in a BTx623 x IS3620c RIL population and verified using GWAS in a sorghum diversity panel (Casa *et al.* 2008). GWAS, a complement to linkage mapping, also suggested several additional putative loci for each of the traits (BD: on Chr. 2, 3, 9; MD: on Chr. 9; RD: on Chr. 9, SWC: on Chr. 1, 6, 9; LWC: on Chr. 6), most of which are supported by a prior research (Shiringani *et al.* 2010). The observations of both co-localized and non-overlapping loci affecting stem diameter traits suggest that stem widths at different heights (base, middle, rachis) share some common genetic determinants, but also have some distinct genetic influences. Besides, co-localizations of stem diameter and water content traits with a number of other bioenergy traits, including plant height, flowering time, branching, stem volume and the ‘dry’ trait, suggest that their inheritance may be functionally (pleiotropy) or physically (linkage disequilibrium) linked.

Combining the results of GWAS and QTL mapping, as is done in this paper, may mitigate the tendency of GWAS alone to find false positive associations. Since QTL found in bi-parental populations are seldom false positives, associations supported by both QTL and GWAS analyses are likely to be true positives. However, such analyses are still prone to false negatives, *i.e.*, not finding true marker-trait associations, for various reasons. First, not all genetic variations can be found in one bi-parental populations, so some GWAS signals need validation from other studies. Second, over-correction of population structure in GWAS may lead to false negatives. Moreover, some variants may be cofounded with population structure, making it difficult to discover the real functions of those variants. All those limitations may impact the power of this work in finding true variants for traits of interest.

Increasing knowledge of the genetic control of stem diameter and water content traits, and identification of corresponding genes and their functions, may lead to tools and strategies for either enhancing or suppressing these traits, supporting general knowledge of plant growth and development, with specific application toward genetic improvement of cultivars to produce biomass for biofuel production.
